# Phospholipase C Zeta in Human Spermatozoa: A Systematic Review on Current Development and Clinical Application

**DOI:** 10.3390/ijms25021344

**Published:** 2024-01-22

**Authors:** Alessandra Parrella, Llanos Medrano, Jon Aizpurua, María José Gómez-Torres

**Affiliations:** 1 IVF Life, Reproductive Medicine, 03540 Alicante, Spain; a.parrella@ivf-spain.com (A.P.); ll.medrano@ivf-spain.com (L.M.); j.aizpurua@ivf-spain.com (J.A.); 2 Cátedra Human Fertility, Facultad de Ciencias, Universidad de Alicante, 03690 Alicante, Spain; 3 Departamento de Biotecnología, Facultad de Ciencias, Universidad de Alicante, 03690 Alicante, Spain

**Keywords:** PLCζ, PLCZ1, human, human spermatozoa, human oocytes, human infertility, human oocyte activation

## Abstract

During fertilization, the fusion of the spermatozoa with the oocytes causes the release of calcium from the oocyte endoplasmatic reticulum. This, in turn, triggers a series of calcium ion (Ca^2+^) oscillations, a process known as oocyte activation. The sperm-specific factor responsible for oocyte activation is phospholipase C zeta (PLCζ). Men undergoing intracytoplasmic sperm injection (ICSI) with their spermatozoa lacking PLCζ are incapable of generating Ca^2+^ oscillation, leading to fertilization failure. The immunofluorescence assay is the most used technique to assess the expression and localization of PLCζ and to diagnose patients with reduced/absent ability to activate the oocytes. In these patients, the use of assisted oocyte activation (AOA) technique can help to yield successful ICSI results and shorten the time of pregnancy. However, the production of a stable PLCζ recombinant protein represents a new powerful therapeutic approach to treating individuals with this condition. We aim to conduct a systematic review focusing on the expression, level, and localization of PLCζ, discussing the novel genetic mutation associated with its impairment. In addition, we highlight the benefits of AOA, looking at new and less invasive methods to diagnose and treat cases with PLCζ dysfunction.

## 1. Introduction

Nowadays, infertility affects 8–12% of couples worldwide [1]. Of these, 20–30% can be attributed to the male, 20–30% to the female, 25–40% are related to both partners while 10–20% remain unexplained [2]. With the advancement of Assisted Reproductive Technologies (ART), such as ICSI and IVF, many couples with infertility issues can achieve pregnancy. ICSI is the most widely used procedure and represents a gold-standard technique to treat male infertility, particularly in cases with oligo-, astheno-, and teratozoospermia or with these combined factors. Additionally, ICSI can address issues related to the zona layer of the oocytes, such as zona pellucida and oolemma/ooplasmic abnormalities, or in other cases, such as elective oocyte cryopreservation, can address low oocyte maturity and recurrent polyspermy [3]. As a result, this technique now constitutes about two thirds of treatments while the IVF technique is used by only one third. Although IVF is an effective technique for many couples with infertility problems, its use is declining due to the high rate of total fertilization failure (TFF), which represents 5–10% of cases. This event, where oocytes fail to fertilize, remaining in metaphase II, occurs in 2–4% of cases when the ICSI technique is carried out and the main cause is largely believed to be oocyte activation deficiency (OAD) [1,4]. The key factor responsible for triggering oocyte activation is a sperm-specific factor, called phospholipase C zeta (PLCζ), which is localized in the perinuclear theca and promotes a series of Ca^2+^ oscillations in the oolemma that are crucial for the resumption of oocyte meiosis, pronuclear formation, and early embryo development [5]. The first study to prove the principal role of PLCζ in the oocyte activation events was conducted by Yoon et al. who demonstrated that spermatozoa with PLCζ deficiency in men undergoing the ICSI procedure were incapable of initiating the intracellular Ca^2+^ oscillation in the oocytes, leading to fertilization failure (FF) [6]. Since then, several studies have provided evidence of a strong association between impaired PLCζ functional ability and male infertility [7]. For instance, a study utilizing quantitative immunofluorescence analysis on a single sperm cell showed that the expression of PLCζ is diminished in infertile men when compared to fertile controls [8]. Furthermore, the same research team found a significant correlation between the total level and localization pattern of spermatozoa exhibiting PLCζ and the fertilization rate in men undergoing ICSI cycles [9]. The first genetic connection between OAD and PLCζ was documented by Heytens et al. with the discovery of a substitution mutation in an infertile male. This mutation was found at position 398 of the PLCζ open reading frame (ORF) in the Y domain, resulting in a histidine to proline substitution (PLCζ ^H398P^) and leading to a disruption of the local protein fold [10]. Kashir et al. identified a secondary mutation within the X domain, involving the substitution of histidine with leucine at position 233 of the open reading frame (ORF). This mutation, designated as PLCζ ^H233L^, is similarly accountable for the disruption of the local protein fold [11]. Recently, two more novel PLCζ mutations have been identified. One mutation, p.I489F, was identified in two infertile brothers and is located in the C2 domain. The other, p.S500L, a missense mutation, was observed in patients experiencing fertilization failure [12]. The current assessment to evaluate the expression of PLCζ is a quantitative immunofluorescence analysis. Kashir and colleagues have shown that there is a considerable variation in the total level of PLCζ not only between infertile and fertile men but also within different ejaculates of the same individual [8]. More tests have been developed to analyze the sperm activation potential in patients experiencing fertilization failure including heterologous and homologous assays such as the mouse oocyte activation test (MOAT) and the human oocytes Ca^2+^ analysis (H-OCA), respectively. These tests clarify the gamete responsible for OAD allowing a more specific and targeted treatment to achieve a higher fertilization rate in subsequent cycles, reducing the need for prolonged and potentially less successful interventions. Indeed, if the cause of fertilization failure is oocyte-related, a modified superovulation protocol should be applied. However, in the case of sperm-related OAD, the most suitable treatment option appears to be AOA [3]. The commonly employed method for AOA involves the use of chemical activating agents such as ionomycin and calcimycin, which are able to trigger the meiotic resumption by raising intracellular Ca^2+^ levels. It has been shown by a variety of studies that, in couples with a history of <30% fertilization rate, the adoption of AOA in the subsequent ICSI cycle improves fertilization and pregnancy rates [3,13,14].Nevertheless, numerous laboratories are investigating the use of human recombinant PLCζ protein as a potential alternative for oocyte activation in cases of PLCζ impairment. Given this background, this study aimed to conduct a systematic review offering a comprehensive bibliometric and bibliographic analysis of publications on PLCζ. The primary focus is on exploring the relationship between PLCζ and human male infertility, delving into the expression, localization, and levels of this protein and discussing novel genetic mutations associated with PLCζ impairment. Additionally, we highlight the benefits of AOA, examining new and less invasive methods to diagnose and manage cases with PLCζ dysfunction. Treating the PLCζ deficit directly addresses a key factor in the complex process of fertilization, offering a targeted solution to enhance reproductive health and increase the chances of successful conception.

## 2. Results

### 2.1. The Compilation of Relevant Bibliographic Sources

**Figure 1:**
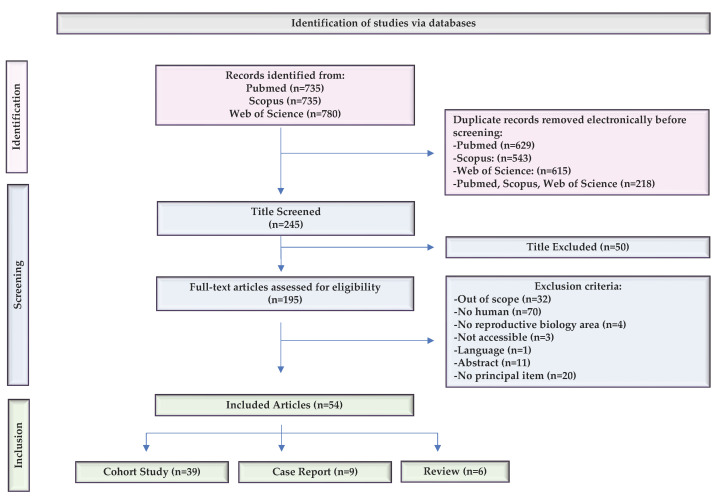
Flow diagram describing the selection process of the articles included in the present review. Template adapted from the PRISMA group [15].

The literature search was conducted by entering keywords in PubMed, Scopus, and the Web of Science. The number of articles obtained in each database was 735 in PubMed, 735 in Scopus, and 780 in the WOS, resulting in a total of 2250 articles. After removing duplicates, the total number of articles was reduced to 245. During the first screening, 50 articles were removed when the title indicated that the topic was not related to PLCζ in spermatozoa. Subsequently, we applied exclusion criteria and discarded 141 articles. Finally, we identified a total of 54 articles that were eligible for this study, which represented 2.4% of all articles initially found. Of these, thirty-nine were cohort studies (72.2%), nine were case reports (16.6%), and six were reviews (11.1%) (Figure 1).

### 2.2. Bibliometric Analysis

**Figure 2:**
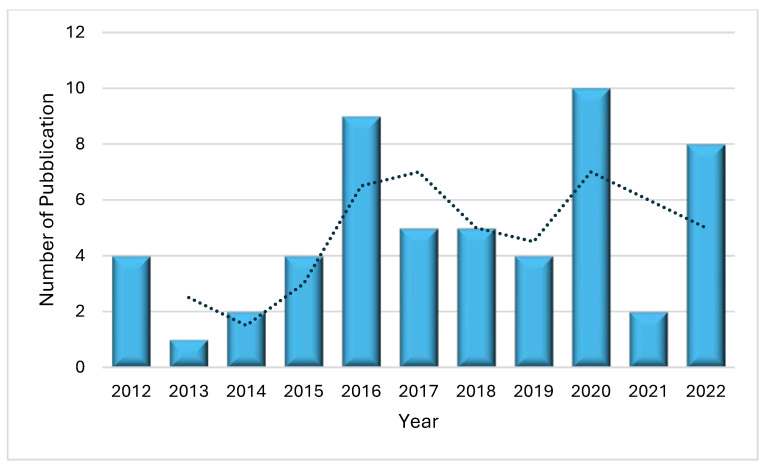
The number of publications on PLCζ in human spermatozoa as a function of the year (2012–2022). According to the trend, the most prolific year was 2020, with 10 publications, followed by 2016, with 9 publications.

**Figure 3:**
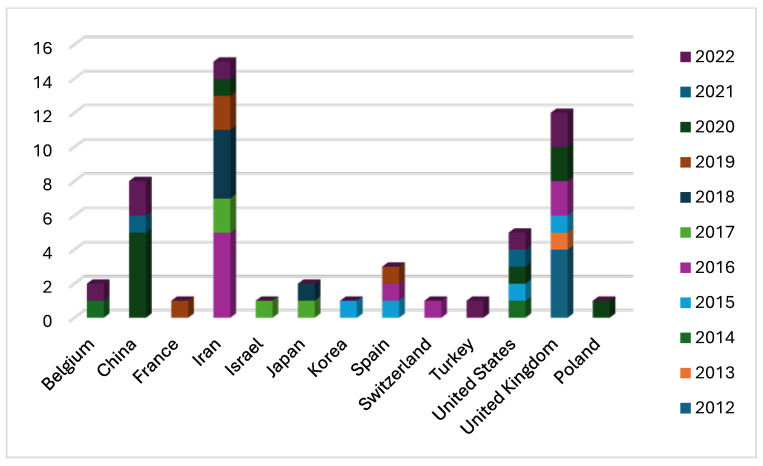
The number of publications on PLCζ in human spermatozoa from different countries as a function of the year (2012–2022). The country with the highest number of publications is Iran, followed by the United Kingdom and China.

The article search was carried out from 2012 to 2022. The analysis of 54 articles revealed that the highest number of publications on this topic was in 2020 with 10 articles (18.5%). In 2016, the number of published articles was nine (16.6%) and slightly decreased in 2022 with eight publications (14.8%). Conversely, the lowest number of articles published on this topic was in 2013 when only one article was published (1.8%) (Figure 2). Probably the variable and not increasing number of articles over the years is not due to a lack of interest in the subject but to the complexity of the study. In fact, the protocol used in the laboratories is not easily applied due to the lack of specificity between the antibody and PLCζ, which leads to a difficult visualization of the results [4]. However, several laboratories worldwide have studied PLCζ in human spermatozoa and based on the information from the first author of the articles, we note that 27.7% (15/54) of publications come from Iran, followed by the United Kingdom with 22.2% (12/54). Nevertheless, China and the United States have also conducted research on this topic albeit to a lesser extent with 14.8% (8/54) and 9.2% (5/54) of publications, respectively (Figure 3).

### 2.3. Bibliographical Analysis

The search carried out in the last 10 years shows 54 articles focused on PLCζ in human spermatozoa. These studies aimed to analyze the expression, localization patterns, and protein level of PLCζ in infertile men and its correlations with sperm characteristics and total/poor fertilization failure. Additionally, studies have investigated the genetic aspects of PLCζ, aiming to identify novel mutations that could impact reproductive outcomes. Furthermore, these articles describe the role of AOA in spermatozoa with a PLCζ deficit and the implementation of new approaches to treating this type of male infertility.

### 2.4. The Analysis of PLCζ

#### 2.4.1. The PLCζ Mechanism of Action

**Figure 4:**
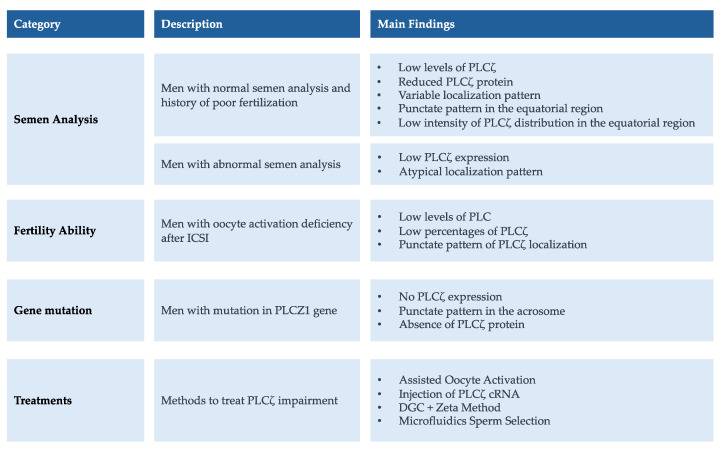
A concise overview of the main findings regarding PLCζ expression and localization in human spermatozoa, complemented by insights into therapeutic strategies.

PLCζ is a cytosolic sperm protein of 70 kDa with the unique ability to promote oocyte activation. It is composed of X and Y catalytic domains between which lies the XY-linker domain, four tandem EF-hand regions, and a C2 domain. The X and Y catalytic domains support the hydrolysis of phosphatidylinositol 4,5-biphosphate (PIP_2_) while the high Ca^2+^ sensitivity is mostly due to the Ca^2+^ binding motifs EF-hands, which enable PLCζ to be active at basal Ca^2+^ levels in the egg cytoplasm. The XY-linker has a net positive electrostatic charge and therefore is involved in the interaction of PLCζ with the negatively charged substrate, PIP_2_-containing membranes. Lastly, the C2 C-terminal domain of PLCζ is crucial for the function of PLCζ involved in targeting proteins to cell membranes. It has been shown that although the deletion of the C2 domain results in a partial loss of enzymatic activity, it has no impact on the enzyme’s Ca^2+^ sensitivity in vitro [16]. The mechanism of PLCZ signaling begins with the PLCζ diffusion in the ooplasm after the fusion of spermatozoa within the oocytes. Here, PLCζ binds intracellular vesicles containing PIP_2_ and its hydrolysis results in the generation of inositol-1,4,5-triphosphate (InsP_3_) and diacylglycerol (DAG). InsP_3_ interacts with its receptor on the surface of the endoplasmic reticulum and promotes the release of calcium [7]. Infertile patients with PLCζ deficiency in their sperm are very likely to encounter IVF/ICSI failure. The quantitative analysis of PLCζ through immunofluorescence is a diagnostic marker to predict sperm oocyte activation capability. However, its clinical use is not always easy to apply because the inadequate specificity between the polyclonal nature of the antibodies and the masked PLCζ antigen causes poor PLCζ visualization and variability in the results. Various laboratories have tried to develop new protocols based on pepdite-blocking experiments [1]. Kashir et al. have proposed a new protocol to improve the clinical visualization of PLCζ using antigen unmasking/retrieval (AUM) protocols, characterized by the addition of HCL or acid Tyrode’s solution or heat. All of the three methods of AUM improved the visualization efficacy for PLCζ compared to that without AUM [17]. However, Meng et al. claimed that this method was not more efficient in visualizing the PLCζ in human spermatozoa when compared with their in-house protocol [4]. Nevertheless, when the protocol is successfully carried out, the PLCζ assay is a good prognostic indicator of OAD. A variety of evidence highlights that the levels, localization patterns, and proportion of human sperm exhibiting PLCζ significantly correlate with ICSI fertilization rates. The expression and localization of PLCζ have been studied in men with normal and abnormal semen parameters, in infertile patients with a history of low, total fertilization failure, recurrent pregnancy loss, and in globozoospermic men (Figure 4).

#### 2.4.2. PLCζ Level, Expression, and Localization in Relation to Semen Analysis

The correlation between semen parameters and PLCζ level, expression, and localization has been described in 23 articles (Table 1). The characteristic localizations of PLCζ are in the equatorial, acrosome, post-acrosomal, and tail region of the spermatozoa, or a combination of these locations. However, the equatorial region is the most dominant localization [1]. A study has demonstrated that in men with normal semen parameters and a history of poor fertilization, the levels of PLCζ were low. The localization pattern was variable among these men, showing a low-intensity PLCζ distribution in the equatorial region, discontinuous patches along the equatorial band, and reactivity on the base of the head [18]. Similar results were found in a case report study in normozoospermic men with a history of complete fertilization failure. The PLCζ levels were decreased in the patient compared to the control, displaying an alteration of the localization of PLCζ. Indeed, the patient showed a significantly low percentage of spermatozoa with a localization pattern in the acrosome and the equatorial region but a significantly higher percentage in the midpiece. Also in the reacted acrosome, a significantly higher percentage was reported in the midpiece [19]. The same research group compared donors to normozoospermic patients and concluded that there was not a significant difference in the distribution patterns of PLCζ. A significant difference was seen when comparing donors to non-normozoospermic men, with a higher PLCζ acrosomal staining observed in donors. When concentration and motility were compared to PLCζ distribution patterns in donors and patients, no correlation was found between them [20]. Nevertheless, Chithiwala et al. have investigated the level of PLCζ in a normal-appearing sperm through Western blot, immunofluorescence analysis, and the PLCζ bioreactivity via an in vitro model of Ca^2+^ release. The spermatozoa had reduced PLCζ protein and bioreactivity showing levels 40–80% lower than control sperm, leading to impaired Ca^2+^ oocyte activation during ICSI. In addition, PLCζ was either absent or present in the post-equatorial region, exhibiting a punctate pattern rather than the typical uniform band in the equatorial region [21]. Investigating the spermatozoa from oligoasthenoteratozoospermic men, low PLCζ expression was seen compared to the control group. The data in this study report a correlation between the sperm parameters and the PLCζ expression. In these patients, the localization pattern displayed an atypical distribution, primarily in the post-acrosomal region, while in the control it was mostly in the equatorial region of the spermatozoa [22]. Tavalaee et al. demonstrated that men with abnormal semen parameters had a significantly lower mean value of PLCζ compared to men with normal semen parameters. A strong correlation was seen between the percentage of PLCζ positive spermatozoa and sperm concentration, motility, and abnormal morphology [23]. The association between PLCζ and sperm morphology was confirmed in a successive study where 23 polymorphic teratozoospermic men were found to have a significantly lower expression of PLCζ compared to 13 normozoospermic men. However, no significant difference in localization patterns and the proportion of PLCζ-expressing sperm were seen [24]. When unexplained and asthenoteratozoospermic men were analyzed and compared to a fertile control, the PLCζ expression was significantly reduced in the cohort study whereas no significant difference was observed in terms of PLCζ localization patterns [5]. Also, Kashir et al. performed a study on male infertility, characterizing PLCζ levels and localization patterns in relation to semen parameters. The study found significant positive correlations between sperm count and the simultaneous presence of the acrosomal and equatorial localization patterns (acrosomal + equatorial) of PLCζ. Additionally, there was a negative correlation with a novel dispersed pattern of localization, not confined to a specific location. When considering sperm motility, there was a positive correlation with the equatorial pattern and a negative correlation with a dispersed pattern of localization. In cases of successful fertilization, higher PLCζ levels, accompanied by a higher prevalence of acrosomal + equatorial patterns were found compared to cases resulting in fertilization failure. This pattern suggests positive indicators for sperm health. It is important to note that the variability in PLCζ levels is linked to diminished sperm health and the presence of dispersed PLCζ is identified as an indicator of reduced sperm viability. These findings underscore the complexity of PLCζ dynamics in relation to both successful fertilization and the overall health and viability of sperm [25]. Besides the association between PLCζ and sperm parameters, it has been investigated if there was any correlation between PLCζ and male age, as well as between PLCζ and sperm quality, focusing on protamine status, DNA oxidation, and fragmentation. A study conducted on 71 men aged between 22 and 54 years revealed no correlation between male age and PLCζ levels and localization but showed a negative correlation between patient age and sperm motility [26]. However, a study in unexplained and asthenoteratozoospermic men revealed that while the age of male subjects did not correlate with PLCζ expression, the PLCζ localization in post-acrosomal, equatorial, and acrosomal + post-acrosomal + equatorial patterns was associated with male age. This suggests that the ageing process might not impact PLCζ protein expression. Instead, it appears to influence specific localization patterns, potentially contributing to fertility-related outcomes in older patients [5]. A study evaluating donor samples reported no correlation between the immunoreactivity of PLCζ and the donor’s age, sperm concentration, motility, or normal morphology. Looking at the sperm quality, no correlation was found with the DNA fragmentation index. Nevertheless, higher sperm DNA oxidation status may be associated with a lower expression of PLCζ [27]. On the other hand, a subsequent study described a negative correlation between DNA fragmentation and the percentage of sperm expressing PLCζ along with a positive correlation between this letter and the fertilization rate. However, no correlation was seen between the percentage of PLCζ and embryo quality or pregnancy rate [28]. A comparison between fertile men with unexplained and asthenoteratozoospermic men found that the only test that was significantly correlated with PLCζ expression was the hyaluronic acid-binding assay because the incomplete formation of the plasma membrane during the maturation process results in a reduced number of hyaluronic acid receptors on the surface of spermatozoa [5]. The levels of PLCζ have also been investigated in men suffering from varicocele. In 35 men with grade II and III of varicocele, the expression of PLCζ was evaluated at messenger RNA and protein levels via real-time PCR and Western blot analysis, respectively. The mean relative expression of PLCζ was significantly decreased in men with varicocele compared to fertile men at both RNA and protein levels. In addition, the DNA fragmentation assessed by SCSA was higher in these patients compared to the control. Given that RNA and DNA are the common targets of ROS, which are elevated in men with varicocele, it is not surprising if both PLCζ expression and DNA fragmentation are compromised [29]. The level and expression of PLCζ have also been evaluated and compared between different sperm selection techniques. A study evaluated the level of PLCζ via flow cytometry in 10 normozoospermic men using fresh samples and density gradient centrifugation (DGC) in incapacitated, capacitated, and acrosome-reacted conditions. PLCζ was significantly higher in samples processed with DGC compared to fresh samples while no difference was seen between DGC samples and acrosome-reacted sperm. The author claims that DGC can remove spermatozoa with a low ability to induce oocyte activation, selecting only the most capable spermatozoa [30]. Khakpour et al. investigated the expression of PLCζ in two sperm selection procedures, DCG and the Zeta potential method using flow cytometry. The results show that PLCζ was higher in DGC samples compared to washed samples and the Zeta method. However, the intensity of PLCζ was significantly higher in samples processed using the Zeta method. Therefore, it seems that the spermatozoa with the best ability to induce calcium oocyte activation are derived from a combination of DGC and Zeta methods [31].

#### 2.4.3. The Level, Expression, and Localization of PLCζ and Fertilization Ability

Quantitative immunofluorescence analysis shows that total levels of PLCζ were significantly higher in fertile patients compared with infertile men diagnosed with recurrent ICSI failure. The localization pattern observed in each control and patient sample was significantly different. Spermatozoa from patients with OAD exhibited a punctate pattern of PLCζ localization, in contrast to the characteristic band observed in the equatorial, acrosome, and post-acrosome regions of spermatozoa in fertile men. Interestingly, total levels of PLCζ displayed a significant variance with the control showing levels like OAD patients [8]. Ferrer-Vaquer et al. also observed significant differences in PLCζ levels among control samples. Specifically, their analysis of PLCζ expression and localization in patients with low (<20%) or TFF revealed significantly lower levels of PLCζ compared to controls. It is worth noting that some control samples also exhibited levels similar to patients with FF, a trend that was similarly observed among the patients. They also examined sperm cells based on their acrosomal status, including intact acrosome, reacted acrosome, and unlabeled acrosome cells. In intact acrosome cells, PLCζ localization was primarily in the acrosomal region of most spermatozoa, with less extent in the equatorial or post-acrosomal regions. On the other hand, in cells with a reacted acrosome or with an unlabeled acrosome, PLCζ protein was displayed only in the post-acrosomal region [20]. The fertilization rates of ICSI and IVF procedures have been compared with PLCζ level and localization. Although no significant difference in PLCζ level and localization was observed in men undergoing IVF cycles compared to control, patients undergoing an ICSI procedure showed a significantly lower total level of PLCζ, with a lower percentage of total spermatozoa exhibiting PLCζ in the post-acrosomal and equatorial region and in the acrosomal + post-acrosomal + equatorial regions [9]. Indeed, another study confirmed that 15 infertile patients with a history of ICSI fertilization failure had lower levels and percentages of PLCζ compared to the fertile men [32]. Different results were found in a recent study that investigated the ratio and mean fluorescence intensity of PLCζ in patients with fertilization rates of ≤40% and ≥60%. The quantitative analysis via flow cytometry showed no significant difference between the two groups [33]. Moreau et al. conducted an intriguing study on the expression of PLCζ in cryopreserved spermatozoa. The cryopreservation process negatively impacts the expression of PLCζ because of the membrane’s damage after cryopreservation. In addition, the author claimed that the post-acrosomal localization, which has a high correlation with fertilization rate, was significantly higher before the cryopreservation [34].

#### 2.4.4. PLCZ1 Mutation Identified in Infertile Males

PLCζ mutations are reported in 14 articles (Table 2). The human PLCZ1 gene is composed of 15 exons and it is situated on chromosome 12. In 2009, Heytens et al. first identified a genetic connection between OAD and PLCζ, pinpointing a substitution mutation in normozoospermic men [10]. The mutation, a histidine to proline substitution, arises in a Y domain of the active side of PLCζ at position 398 of the open reading frame (PLCζ ^H398P^), resulting in the abolishment of the hydrolytic activity of PLCZ1 protein and the inability to generate Ca^2+^ oscillation in the ooplasm [1]. Subsequently, in the same patient, Kashir et al. identified a second mutation in the X domain resulting in a histidine to leucine substitution at position 233 of the open reading frame (PLCζ ^H233L^), involved in the disruption of the local interaction within protein folding. This mutation does not eliminate PLCζ’s ability to generate Ca^2+^ oscillation, but its function is compromised. The author revealed that both mutations were heterozygous, with PLCζ ^H398P^ being inherited from the patient’s father and PLCζ ^H233L^ being inherited from the mother. This discovery was significant as it demonstrated for the first time that PLCZ1 can be inherited maternally, and that this could result in a loss of sperm function in the son and subsequently in infertility [11]. To verify the bilateral inheritance of the two mutations, the author analyzed the distribution of PLCζ ^H398P^ and PLCζ ^H233L^ as well as the localization patterns of fluorescent mutant PLCζ isoforms in human embryonic kidney cells (HEK293T) obtained from an infertile man with known PLCζ ^H398P^ and PLCζ ^H233L^ mutations. These mutations, located on different chromosomes, exhibit independent inheritance and are never present at the same time. Consequently, spermatozoa can only carry either the PLCζ ^H398P^ mutation or the PLCζ ^H233L^ mutation, but not both mutations simultaneously [35]. Several studies have tried to investigate the PLCζ mutations in men who experienced TFF. Escoffier et al. studied two infertile brothers with complete fertilization failure after ICSI. Whole-exome sequencing indicates a missense homozygous mutation in PLCZ1, c.1465A > T; p.Ile489Phe, converting Ile 489 into Phe. To understand the effect of this mutation, the PLCζ expression and localization were examined. Most of the spermatozoa did not display the PLCζ staining and a few of them displayed a faint punctuate staining over the acrosome, instead of showing the classic band. The absence of the PLCζ was confirmed with Western blot, where no reactivity on the patients’ lane was observed. Therefore, this mutation causes the absence of the protein in the sperm, leading to reduced Ca^2+^ oscillation and ultimately a lower rate of oocyte activation and early embryonic arrest [36]. Another important gene involved in actin polymerization during acrosomal biogenesis, the formation of sperm head morphology, capacitation, and the acrosome reaction is CAPZA3 [capping protein (actin filament) muscle Z-line, alpha 3], which is localized back-to-back with PLCζ and with which it shares a common bidirectional promoter with a putative cAMP responsive element modulator of the protein recognition site. Considering this, a study explored if there is an association between these two genes by investigating their expression in 59 infertile patients with total, low, and high fertilization rates after ICSI. The results showed a significant correlation between the relative expression of PLCζ and CAPZA3 and between the fertilization rates and these two genes. Notably, men with low fertilization rates exhibited a significantly reduced expression of both genes. Furthermore, a mutation within the predicted promoter of CAPZA3 was discovered in an individual who had a low expression of both PLCζ and CAPZA3 genes. The promoter region in question is known to bind with a testis-specific dimeric DNA-binding protein named human regulatory factor X4. These results suggest that CAPZA3 may serve as a useful marker for assessing spermatozoa’s ability to initiate oocyte activation [37]. In a study, 37 patients diagnosed with oocyte activation failure (OAF), were segregated into two groups: the first group had FF due to defects in oocyte activation (OAF, n  =  22), while the second group (n  =  15) experienced FF due to other causes (“no-OAF”, n  =  15). Samples from 13 men with good fertilization (fertilization rate > 50%) were used as controls. Compared to the no-OAF group where only one patient had a mutation, all of the patients of the OAF group carried at least one mutation in the PLCZ1 coding sequence. Of the six mutations identified, five of them were single-nucleotide missense mutations: p.I120M, located at the end of the EF-hand domain; p.R197H, p.L224P, and p.H233L, located at the X catalytic domain; and p.S500 L, located at the C2 domain. The sixth mutation, a frameshift variant (p.V326K fs * 25), generates a truncated protein at the X-Y linker region. This mutation is responsible for the FF probably due to unknown factors acting in downstream events. Interestingly, PLCZ1 protein localization and expression levels in sperm did not differ across groups [12]. Successive studies identified novel mutations in men who experienced TFF. In four couples with a history of fertilization failure, five novel mutations in PLCZ1 were identified using whole-exome sequencing and Sanger sequencing. In two patients a homozygous nonsense mutation c.588C > A (p.Cys196∗) and homozygous missense mutation c.590G > A (p.Arg197His) were found. The other two patients showed compound heterozygous mutations: one had a heterozygous mutation c.588C > A (p.Cys196∗) and the missense heterozygous mutation c.1259C > T (p.Pro420Leu), while the other one had compound heterozygous frameshift mutations c.972_973delAG (p.Thr324fs) and c.1234delA (p.Arg412fs) [38]. In a separate investigation, a Chinese patient was found to possess the heterozygous mutations c.1259C > T (p.P420L) and c.1733T > C (p.M578T), while in another patient, a novel homozygous mutation, c.1727T > C (p.L576P), was identified. All three of these mutations have been demonstrated to hinder the hydrolytic activity of PLCζ, resulting in an impairment of the protein [39]. The same author reported a novel missense homozygous mutation in PLCζ, c.1658 G > C; p. R553P, which leads to the conversion of arginine 553 to proline. This mutation does not compromise the production of the protein, but the microinjection of the mRNA transcribed from the PLCζ R553P mutation gene was unable to trigger oocyte activation and embryo development [40]. Subsequently, another three novel homozygous variations in PLCZ1 have been detected in patients with TFF. A novel nonsense variation, c.C588A (p.C196X), was identified in one patient, while two novel missense variations, c.T1048C (p.S350P) and c.C736T (p.L246F), were found in two patients. To investigate the effect of these variations on PLCζ localization, immunofluorescence staining was carried out on both patients and donors. In the control group, PLCζ displayed localization at the equatorial region. However, patients with the nonsense variation p.C196X exhibited no expression of PLCζ. In contrast, patients with the missense variation p.S350P showed that most spermatozoa had PLCζ localization in the post-acrosomal region, whereas, in patients with p.L246F, PLCζ was localized in the equatorial region [41]. In the same year, six novel mutations were discovered in five patients with TFF using Sanger sequencing. The following mutations were identified: two patients had a missense mutation c.1151C > T (p.A384V) and a homozygous nonsense mutation c.588C > A (p. C196*), already reported in a previous study [38]. The other three patients carried compound heterozygous mutations: one patient had nonsense mutation c.588C > A (p.C196∗) and missense mutation c.830T > C (p.L277P), the other one showed a 3 bp in-frame deletion c.1129_1131delAAT (p.N377del) and missense mutation c.1733T > C (p.M578T), while the last patient reported the splicing mutation c.570 + 1G > T and the missense mutation c.1344A > T (p.K448N). To further investigate the effect of these mutations, PLCZ1 protein levels were evaluated using Western blot on semen samples collected from five patients with mutations and five control donors. While protein levels of PLCZ1 were detected in all of the controls, spermatozoa from the five patients with homozygous or compound heterozygous mutations had almost undetectable levels [42]. In a case study, a novel homozygous PLCZ1 nonsense mutation, c.588C > A (p.Cys196Ter) has been found in an infertile man with FF. The mutation produces a loss of function of the gene because it introduces a premature termination codon in PLCZ1 mRNA. Indeed, no PLCZ1 protein was identified in the sperm from the patient using Western blot and immunofluorescence analysis [43]. Studies have demonstrated that the transcriptome profile differs not only between fertile and infertile men but also among men with abnormal semen parameters. In a recent study, the RNA profile of 44 selected genes of spermatozoa coming from patients with severe oligozoospermia was compared to those of normozoospermic men. The genes involved in embryo development including FOXG1, PLCZ1, POU5F1, STAT4, and TOP2A were identified only in the normozoospermic group. The authors proposed that the absence or low expression of two genes, PLCZ1 and POU5F1, both critical for embryo development, could be linked to fertilization failure and embryo arrest at the eight-cell stage [44]. Moreover, another study has investigated the gene expression of 105 asthenozoospermic men, revealing the discovery of a homozygous missense mutation in SLO3 in one patient. Looking at the morphology of the spermatozoa, a high rate of head malformations was seen in the acrosome region; the sperm displayed swollen or short and irregular width midpieces, coil-shaped flagella, small acrosome, and an absence of acrosome. Unlike the control, where the PLCζ was localized in the acrosomal region of the spermatozoa, in the patient PLCζ was absent or displayed at the base of the neck and middle piece of the tail. This indicates that the localization of PLCζ is influenced by the SLO3 deficiency [45]. Similarly, in a case study, a patient diagnosed with globozoospermia was found to have a homozygous missense variant (c. 3671G > A) in the sperm specific antigen 2 (SSFA2). After the failure of oocyte activation in 24 MII oocytes retrieved during an ICSI cycle, the expression of PLCζ was analyzed and found to be significantly lower than in the control group. The OAF caused by the PLCζ defect was resolved using ICSI with AOA, and a healthy baby was delivered after the transfer of the embryo on day 3. Therefore, this treatment has the potential to rescue the SSFA2 variant [46].

#### 2.4.5. PLCζ in Globozoospermic Patients

Globozoospermia is a rare male infertility disorder in which spermatozoa have round heads, abnormal or absent acrosomes, and are often defective in two genes, DPY19L2 and SPATA16 [2]. In patients with globozoospermia, fertilization failure is caused by the absence or deficit of sperm oocyte activation factors such as PLCζ, as reported in five articles (Table 3). Indeed, studies have shown that these individuals have a lower PLCζ mRNA, and protein level compared to fertile men [47,48]. This finding was also confirmed in a study of 32 globozoospermic men with DPY19L2 deletion, in which both the RNA and protein levels of sperm PLCζ were significantly lower than those in fertile men [49]. There are two subtypes of globozoospermia, the complete form (CG) and the partial form (PG), which are distinguished by the percentage of spermatozoa with round-headedness and acrosomal abnormalities. The complete form is the most severe and is characterized by 100% of spermatozoa having a round head and acrosomal hypoplasia; the partial form has a variable proportion of spermatozoa exhibiting round-headedness and acrosomal mark dysmorphism. A study showed that CG men have extremely reduced PLCζ levels, visible in the post-acrosomal region of the spermatozoa, compared to PG men. In addition, genomic and transcriptomic analyses carried out on three CG men showed that the PLCZ1 gene was mutated and underexpressed in all three men [2]. It has been demonstrated that the use of motile sperm organelle morphology evaluation (MSOME) can improve PLCζ levels in globozoospermic men. After the isolation via micromanipulation of the spermatozoa identified through MSOME, the levels and localization partner were evaluated. Control sperm had a significantly higher total level and proportion of spermatozoa exhibiting PLCζ compared to non-MSOME-selected sperm. However, no difference was seen between the MSOME-selected spermatozoa and the control. This indicated that the MSOME is a useful treatment in globozoospermic men to select spermatozoa with higher oocyte activation ability [50].

#### 2.4.6. AOA Treatment in Men with PLCζ Dysfunction

AOA is the most effective approach to treating patients with oocyte activation issues caused by the low expression or abnormal localization patterns of PLCζ. The treatment, detailed in four articles (Table 4), aims to enhance the release of Ca^2+^ from the oocyte endoplasmic reticulum. This, in turn, facilitates achieving a normal fertilization rate following ICSI cycles. AOA can be performed by different methods such as applying an electrical field to the oocytes, aspirating cytoplasm vigorously during ICSI, or using chemical agents. The most common method for promoting intracellular calcium transients in the oolemma via extracellular influx involves the application of chemicals. Among the available agents, calcium ionophores such as ionomycin and calcimycin are the most utilized. Several studies provide evidence supporting AOA’s potential to boost fertilization rates and embryo development, making it a promising option for couples experiencing previous fertilization failures [13]. In a study performed in 2019, patients undergoing ICSI cycles were divided according to the mean percentage of PLCζ. Men with >80% of PLCζ were classified as group 1 and served as control while those with <60% of PLCζ were classified as group 2. Some patients of group 2 had oocytes exposed to AOA, revealing a significant improvement in fertilization rates, although embryo cleavage and quality showed no marked differences compared to the group without AOA [51]. In another study, in infertile men with abnormal sperm morphology and low or total fertilization failure, the level of PLCζ in sperm and/or proportion of spermatozoa exhibiting PLCζ was significantly lower compared to the control. Some patients with a reduction of both parameters underwent an ICSI cycle with AOA, achieving higher fertilization and clinical pregnancy rates [52]. An instance where AOA proves indispensable in addressing male infertility even when PLCζ expression levels are normal arises when a patient’s sperm is implicated in partial hydatidiform moles—an aberrant form of human pregnancy characterized by the excessive proliferation of placental villi or the absence of embryonic development. The majority of such pregnancies result from dispermy, involving the fertilization of oocytes by two sperm. In a detailed case report of a couple experiencing recurrent partial hydatidiform mole pregnancies, it was revealed that 83.3% of in vitro–matured human oocytes, upon injection, failed to exhibit Ca^2+^ release, and 76.9% did not demonstrate normal pronuclear development. Despite PLCζ expression levels comparable to the control, AOA implemented in subsequent ICSI cycles led to successful, healthy deliveries. Therefore, even when PLCζ levels are within the normal range, spermatozoa involved in partial hydatidiform moles are unable to induce Ca^2+^ oscillation, resulting in partial or complete fertilization failure [53]. It is crucial to note that OAD is not always attributed to the sperm factor and therefore it is important to identify the gamete responsible for OAD to determine the most appropriate clinical treatment. Indeed, a recent study involving 76 couples showed that in couples with oocyte-related OAD, successful fertilization, pregnancies, and deliveries were achieved with an adjusted superovulation protocol, while in couples with a sperm-related OAD, AOA was the best approach to optimize the clinical results [3].

#### 2.4.7. Treatment Approach in Men with PLCζ Impairment

While a recent study highlights the absence of a thorough investigation into the efficiency of the AOA procedure, it continues to be the most widely employed technique for couples facing OAD [14]. Nevertheless, researchers are actively exploring alternative treatments to induce Ca2+ oscillations in oocytes, as discussed in eight articles (Table 5). These treatments involve methods such as microinjecting PLCζ mRNA or recombinant protein into human oocytes [7,54]. A study has demonstrated that introducing recombinant hPLCζ in vitro matured MII oocytes, without spermatozoa, results in the formation of one pronucleus and two cells within 48 h. Furthermore, injecting rhPLCζ into unfertilized oocytes after ICSI resulted in the formation of two pronuclei in five out of eight oocytes (62,5%) [16]. Interestingly, the injection of PLCζ cRNA in unfertilized oocytes promotes a substantial initial rise in Ca^2+^ levels, followed by a series of small Ca^2+^ oscillations that gradually increase in frequency. Using particle image velocimetry, it was possible to observe that each transient Ca^2+^ concentration change goes with a small movement of the cytoplasm. These movements are correlated with the exact timing of the Ca^2+^ increase that occurs repetitively after the injection of PLCζ cRNA [55]. Moreover, the pattern of Ca^2+^ oscillation has been analyzed by comparing the injection of PLCZ1 RNA injection with other AOA methods such as cytosolic aspiration, electrical stimulation, and ionomycin treatment. The results demonstrated that the pattern of Ca^2+^ oscillation was comparable to that induced with ICSI. When the timing of PN formation and embryo development rate was analyzed, the embryo coming from the oocyte injected with PLCZ1 RNA had better development than the embryos coming from other methods. This indicates that this technique is a good therapeutic approach to rescuing human oocytes that can fail to activate [56]. However, a stable recombinant human PLCZ1 protein (rhPLCZ), is not commercially available [54]. Although hPLCZ RNA can produce optimal and physiological egg activation, it is yet unknown whether its injection in human oocytes would have any adverse effects [13]. A less invasive and more practicable method has been utilized to increase the selection of spermatozoa having PLCζ. A recent study compared the PLCZ1 expression of patients with fertilization failure following ICSI in spermatozoa selected with microfluidics and DGC methods. The RT-PCR analysis revealed a marked enhancement in the expression of the PLCZ1 gene in the semen sample isolated using microfluidics, as compared to the sample obtained through the DCG method and the unprocessed sample. Furthermore, the utilization of the microfluidics selection led to a higher proportion of top-quality embryos compared to those obtained via the DCG method [57].

## 3. Discussion

PLCζ is a soluble cytosolic sperm factor able to induce oocyte activation via the release of intracellular calcium ions from the endoplasmatic reticulum stores. A wide range of research has been conducted to investigate the role and function of PLCζ in male infertility, considered a causative factor in cases of fertilization failure. Researchers have investigated the PLCζ expression and localization in men with normal and abnormal semen parameters highlighting a strong correlation between sperm concentration, motility, and morphology with the percentage of spermatozoa positive for PLCζ. Notably, the latter plays a significant role in predicting PLCζ expression [22,23,25]. In contrast to these findings, Azad et al. reported no significant differences between polymorphic teratozoospermic patients and the control group in terms of the percentages of sperm that express PLCζ and their localization patterns. However, they did observe a significantly lower level of PLCζ expression among men with polymorphic teratozoospermia [24]. The same author shows a similar trend in oligoasthenoteratozoospermic men who displayed a significantly reduced percentage of sperm expressing PLCζ compared to normozoospermic men [22]. Another study performed by Ferrer-Vaquer et al. found no correlation between sperm characteristics and PLCζ expression in patient and donor samples, demonstrating that PLCζ expression might be independent from motility and concentration [20]. Rahimizadeh et al. recently investigated the possible connections between PLCζ levels and male age, sperm characteristics, DNA integrity, and cellular maturity in spermatozoa from men with either asthenoteratozoospermia or unexplained infertility. Although fertile men showed significantly higher levels of PLCζ than infertile or subfertile men and a correlation between PLCζ and the ability to bind hyaluronic acid was observed, no other associations could be identified [5]. Similar results were seen in another study where PLCζ immunoreactivity was not associated with the donor’s age, sperm concentration, motility, and DNA fragmentation. However, they found an inverse relationship with oxidative status [27]. On the contrary, Tavalaee et al. observed a significant negative correlation between DNA fragmentation and PLCζ expression, indicating that artificial oocyte activation may be required in males with high levels of DNA fragmentation [23]. The pattern of PLCζ localization in spermatozoa is a crucial consideration, as it has implications for both sperm health and the likelihood of reproductive success. A study has revealed that a dispersed PLCζ pattern is linked to lower sperm viability, while the presence of acrosomal + equatorial PLCζ is strongly associated with healthier sperm and successful fertilization. Variations in PLCζ patterns are correlated with declining sperm health, potentially contributing to male subfertility and the effects of advancing male age. Furthermore, in cases of successful fertilization, significantly larger amounts of PLCζ and higher ratios of equatorial and acrosome patterns were observed [25]. These results are consistent with previous studies reporting similar localization patterns [8,26]. A study on oligoasthenoteratozoospermic men showed significantly reduced proportions of the equatorial pattern and its combinations (equatorial + acrosome and equatorial + post-acrosomal) compared to the control group [22]. Interestingly, studies found that the total level, localization patterns and proportion of sperm exhibiting PLCζ are correlated with fertilization rates for the ICSI procedure [9,28]. Indeed, the mean percentage of PLCζ-positive sperm and the level of this protein were significantly decreased in FF patients compared to the control population [32]. A low expression of PLCζ has also been linked to both failed and low success in ICSI fertilization in normal-appearing sperm [18,19].Contrary to this, Aras-Tosun et al. demonstrated that the percentage and mean fluorescent intensity of the PLCζ protein do not exhibit a correlation with low fertilization and clinical pregnancy rates [33]. Nevertheless, there was significant variability in total PLCζ levels among individual controls. While patients with FF showed notably lower overall levels of PLCζ, the test results displayed variability, with certain controls exhibiting levels comparable to those found in FF samples [8,20]. This could be due to the lack of specific antibodies, making its implementation challenging. A novel methodology has been introduced to improve the visualization efficacy of PLCζ. This involves the utilization of an antigen unmasking/retrieval protocol, impacting both the relative fluorescence levels and the proportion of sperm exhibiting detectable PLCζ fluorescence [17]. On the other hand, Meng et al. demonstrated that their in-house method showed superior visualization and reliability to the unmasking/retrieval protocol [4]. Researchers have reported the inability of globozoospermic patients to evoke long-term Ca^2+^ oscillations because their sperm heads are rounded and devoid of the acrosome. These patients have an abnormal punctate pattern of PLCζ localization with some spermatozoa displaying an acrosomal bud. Moreover, the relative expression of PLCζ at RNA and protein levels is significantly lower compared to fertile men and therefore it is not surprising if these men are unable to fertilize naturally [47,48,49]. AOA is considered the best therapeutic option for these patients and for those suffering from OAD. Studies have shown that the addition of a calcium ionophore during the ICSI procedure can improve oocyte fertilization in men with a deficiency of PLCζ in their spermatozoa [21,51,52]. However, if the OAD is not derived from the male gamete, AOA may not always provide benefits. A recent study suggests that OAD can result not only from sperm-related factors but also from oocyte-related factors. Therefore, in cases where fertilization failure is attributed to oocytes, a modified superovulation technique should be considered [3]. Concurrently, in couples with sperm-related problems, various research laboratories are actively investigating new PLCζ-based techniques. Significant emphasis is being placed on sperm selection techniques to augment the percentage of spermatozoa exhibiting PLCζ. For instance, a study found that the use of density gradient centrifugation significantly decreases the proportion of sperm that did not express PLCζ removing the sperm with a low capability to induce oocyte activation [30]. Two sperm selection techniques have shown promise in increasing the expression levels of the PLCζ gene in sorted sperm. Microfluidic sperm selection effectively isolates sperm with elevated PLCZ1 expression levels. Meanwhile, the Zeta method enhances the intensity of PLCζ expression in selected spermatozoa, rather than increasing the number of spermatozoa showing PLCζ as seen with DGC. Therefore, it has been suggested that a good selection of spermatozoa with oocyte activation ability will be achieved with the combination of DCG and the Zeta method [31]. Studies have demonstrated that the MSOME technique can be beneficial in selecting spermatozoa that exhibit acrosome bud morphology and express PLCζ within their head regions. Specifically, up to 43% of spermatozoa with acrosome bud morphology have been found to express PLCζ [50]. Another approach is the microinjection of mRNA or recombinant protein in MII [7]. The injection of PLCZ1 RNA has been shown to induce the same pattern of Ca^2+^ oscillations as seen during fertilization, leading to the development of parthenogenetic blastocysts in humans [56]. However, it remains unclear whether the injection of hPLCZ1 RNA can cause any harm to human oocytes, despite its ability to produce optimal and physiological egg activation [13]. Nonetheless, the microinjection of pure hPLCZ could be a viable strategy for rescuing PLCζ and could be implemented immediately after an ICSI fertilization failure. This approach may be particularly beneficial for couples who have experienced repeated failures and have been advised to consider sperm donation [16]. Studies have found that genetic factors can contribute to PLCζ deficiency in male-related OAD. Many PLCZ1 gene mutations have been found in male individuals presenting low or total FF following the ICSI technique [11,12,35,36,37,38,39,40,41,43,45,46]. Most of these are homozygous PL CZ1 mutations, but it has been discovered that even if the heterozygous mutation is less common than homozygous mutations, they can decrease the overall quantity of functional protein in all sperm and be sufficient to result in FF following ICSI [12]. The impact of these mutations seems to depend on both their location within the PLCZ1 gene and their inheritance manner. It is probable that the deficiency of PLCZ1 is associated with unknown processes that are linked to gene expression or regulation [1]. Indeed, various processes can affect gene expression at the transcriptional and/or translational levels and identifying the specific process that impairs PLC function can be challenging. For instance, only a limited number of studies have examined the bidirectional promoter of PLCZ1, with Javadian-Elyaderani et al. being the only researchers to identify a variant in the CAPZA3 promoter located close to PLCZ1. However, the author hypothesized that this variant may not affect the transcription of PLCZ1 [37]. Further research is needed to fully comprehend the exact role of the bidirectional promoter in relation to gene transcription. Gene expression at the transcriptional and translational levels involves various components, such as RNA polymerase, enhancers, silencers, and RNA molecules, including coding and non-coding RNAs. Investigations should explore the possibility that variations in these elements can compromise PLCZ1 expression, potentially leading to the malfunction of the PLCZ1 protein and male infertility.

## 4. Materials and Methods

### 4.1. Literature Search and Information Processing

We conducted a systematic search of the literature published in PubMed (https://pubmed.ncbi.nlm.nih.gov, accessed on 21 August 2022), the Web of Science (WOS) (https://www.webofscience.com/wos/woscc/basic-search, accessed on 21 August 2022), and Scopus (https://www.scopus.com/, accessed on 21 August 2022) databases between January 2012 and July 2022. While conducting the search in the WOS database, the “search all database” option and the field tag “topic” were utilized, whereas in the Scopus database, the field tag “Article title, Abstract, Keywords” was selected. The search strategy was limited to studies conducted on humans, full-text articles, and published in English. Combinations of the following keywords were used: PLCζ, PLCZ1, human, human spermatozoa, human oocytes, human infertility, and human oocyte activation.

### 4.2. Study Selection and Eligibility Criteria

Microsoft Excel spreadsheet software was used to collect all of the results from the literature search and duplicates were recognized using electronic and manual methods. All studies reporting PLCζ in human spermatozoa were considered eligible for abstract screening. The documents were selected based on two inclusion criteria: (i) studies published in the last 10 years and (ii) studies in humans only. The main exclusion criteria were as follows: (i) studies not focused exclusively on PLCζ in humans, (ii) studies not belonging to the reproductive biology area or (iii) out of scope, (iv) work not published in English, (v) work not accessible or (vi) without an available full text, and (vii) studies where PLCζ was not the main topic (Figure 1). MJG-T and AP chose the inclusion and exclusion criteria. The studies were initially screened based on title, abstract, and content by two authors independently (AP, LM). While inconsistencies or questions were resolved with consensus by mutual discussion among three authors (MJG-T, AP, and LM), the final list of included studies was approved by MJG-T.

### 4.3. Data Extraction

For each study, the following data were extracted: titles, names of authors, year of publication, source title, affiliation, country, language, study type (case study, cohort study, review), and main findings. These data were imported into a standardized data extraction sheet, revised by AP, LM, and MJG-T.

## 5. Conclusions

Over the last decade, research has demonstrated the potential of PLCζ to promote oocyte activation. The PLCζ assay is a crucial tool in diagnosing patients with a deficiency of PLCζ in their spermatozoa, as it helps to identify those who may be at risk for failed fertilization during IVF/ICSI procedures. By identifying those patients, appropriate treatment strategies, such as the use of artificial oocyte activation procedures, can be employed to increase the chances of successful fertilization and embryo development [1,54]. In addition, successfully treating PLCζ-related infertility can directly contribute to better reproductive health, bring hope and relief, and reduce the emotional strain associated with fertility struggles. Various pieces of evidence suggest that the levels, proportion, and localization patterns of PLCζ in human sperm are closely linked to male infertility. The predominant pattern of PLCζ localization is in the equatorial region, often missing in patients experiencing FF [36]. However, some spermatozoa can display different localization either alone or combined with another pattern. Furthermore, studies have also found that the expression of PLCZ1 mRNA in the semen samples of patients with failed fertilization is lower when compared to those with high fertilization rates. Several mutations have been described in the human PLCZ1 gene, but more studies are needed to investigate the different components that can interfere with gene expression at the transcriptional and translational levels such as enhancers, silencers, and RNA polymerase [1]. The impact of these factors on PLCZ1 expression may cause the dysfunction of PLCZ1 protein and therefore male infertility. In the last year, researchers have been focused on targeted therapy for patients with PLCζ deficit. Indeed, various human rPLCZ1 proteins have been developed whose injection generates multiple Ca^2+^ oscillations, like the physiological response to calcium. Despite its potential, the practical application of this approach is not yet feasible due to the need for further interventions to develop a purified and stable molecule that can be used safely. Consequently, AOA remains the best approach but should be used with caution and only in cases of fertilization failure until its safety in addressing other infertility issues is established. As research and medical advancements continue, there is hope for further improvements in treating PLCζ-related infertility, ultimately contributing to better reproductive health outcomes for affected individuals and couples.

## Figures and Tables

**Table 1:** Studies on PLCζ localization, expression, and protein level.

Ref.	PLCζ Features	Population	Sample	PLCζ Identification	Main Findings
[1]	Localization patterns, expression profile, genetics, and identification of PLCZ1 deficit	Infertile patients (N = NA)	NA	NA	The expression and localization of PLCZ1 in human sperm are associated with male infertility. Infertile males showed lower PLCZ1 levels in their sperm than fertile males. PLCZ1 was found to be predominantly expressed in the equatorial region of the sperm but sometimes in combination with other regions.
[4]	Method to identify PLCζ	Recurrent FF, low fertilization, TFF, abnormal sperm head morphology (N = 12)	Fresh	Immunofluorescence	Specificity testing of antibody-antigen binding indicated that the house method showed more specific binding than spermatozoa treated using the antigen unmasking method (AUM), resulting in the highest relative fluorescence intensity.
[5]	Expression profile, localization patterns	Asthenoteratozoospermic (N = 40) and unexplained infertile patients (N = 40)	Fresh	Immunofluorescence, Western blot	PLCζ expression was significantly reduced in unexplained infertile and asthenoteratozoospermic patients compared to fertile men. No significant differences were observed among the experimental groups in terms of PLCζ localization patterns.
[8]	Level, localization patterns	OAD patients with recurrent ICSI failure (N = 5)	Frozen	Immunofluorescence	Control subjects presented a significantly higher proportion of sperm exhibiting PLCζ immunofluorescence compared with OAD men. Total levels of PLCζ in sperm from control and OAD patients exhibited significant variance. Predominant PLCζ localization patterns varied between control and OAD samples with no predictable or consistent pattern.
[9]	Expression profile, localization patterns	Infertile patients (N = 44)	Fresh	Immunofluorescence	Total levels, localization patterns, and the proportion of sperm exhibiting PLCζ are correlated with fertilization rates for ICSI, but not for IVF.
[17]	Method to identify PLCζ	Infertile patients (N = NA)	Fresh	Immunofluorescence	All methods of the antigen unmasking method (AUM) enhanced PLCζ visualization efficacy compared to those without AUM. Examination of sperm from individual donors revealed that AUM differentially affects observable PLCζ fluorescence, and the proportion of sperm exhibiting detectable PLCζ fluorescence in sperm from different males.
[18]	Expression profile, localization patterns	Normozoospermic with failed or low fertilization (N = 4)	Fresh	Western blot and immunofluorescence	Male partners of couples with failed or low success ICSI fertilization but with normal semen analysis parameters showed low expression levels of PLCZ1.
[19]	Expression profile, localization patterns	Normozoospermic with TFF (N = 1)	Fresh/Frozen	Gene sequencing, immunoblotting, immunofluorescence, MOAT, MOCA, AOA	PLCζ expression level and distribution were significantly disrupted despite the normal sperm activation potential in functional testing using mouse oocytes.
[20]	Expression profile, localization patterns, genetics	Low fertilization and TFF(N = 15)	Fresh	Gene sequence, Western blot, and immunofluorescence	The distribution pattern of PLCζ did not vary significantly between donor and patient samples. Levels of PLCζ protein in sperm cells showed an interindividual variability both in patient and donor samples. Several SNPs previously reported in infertile patients were also present in fertile men.
[21]	Expression profile, localization patterns, AOA	Normozoospermic men with fertilization failure (N = 1)	Fresh	Western blot, immunofluorescence, PLCζ bioactivity by an in vitro model of Ca^2+^ release.	Deficiency of PLCζ in normal-appearing sperm was associated with impaired Ca^2+^ -dependent oocyte activation during ICSI. Use of calcium ionophore following ICSI improved embryo developmental competence.
[22]	Expression profile, localization patterns	Oligoasthenoteratozoospermic patients (N = 25)	Fresh	Immunofluorescence	The mean percentage of sperm cells expressing PLCζ was significantly lower in OAT than the control. The localization patten was altered with a predominance in the post-acrosomal region.
[23]	Level	Patient with normal (N = 32) and abnormal (N = 23) semen parameters	Fresh	Flow cytometry	PLCζ was significantly lower in men with abnormal semen parameters compared to those with normal parameters. Significant correlations were observed between sperm concentration, motility, and abnormal morphology with the percentage of PLCζ positive spermatozoa. Negative relationship was observed between the percentage of PLCζ positive spermatozoa and sperm DNA fragmentation.
[24]	Expression profile, localization patterns, mean level	Polymorphic teratozoospermic patients (N = 23)	Fresh	Immunofluorescence, Western blot	Relative PLCζ expression was significantly lower in polymorphic teratozoospermic men, as compared with control men; there was no significant difference in localization patterns and proportion of PLCζ-expressing sperm between polymorphic teratozoospermic patients and control men.
[25]	Expression profile, localization patterns	Infertile patients (N = 65)	Fresh	Immunofluorescence and Immunoblotting	Acrosomal and equatorial PLCζ correlated most to sperm health, while dispersed PLCζ correlated to decreased sperm viability. Total levels of PLCζ exhibited significant correlations with sperm parameters. Significantly higher levels of PLCζ were exhibited by cases of fertilization success, alongside higher proportions of acrosomal + equatorial patterns, and lower levels of dispersed PLCζ.
[26]	Expression profile, localization patterns	Infertile patients (N = 27)	Fresh	Immunofluorescence	While the progressive motility parameter was negatively correlated with male age, the proportion, total level, and localization patterns of PLCζ were not associated with male age.
[27]	Expression profile, DNA fragmentation, oxidation status	Infertile patients (N = 44)	Fresh	Immunofluorescence	The duplicate PLCζ tests on two sperm samples from each patient showed similar results. Immunoreactivity of PLCζ was not associated with donor’s age, sperm concentration, motility, and the percentage of normal form as well as DNA fragmentation index. However, lower expression of PLCζ in sperm may be associated with higher sperm DNA oxidation status.
[28]	Expression profile	Infertile patients (N = 35)	Fresh	Flow cytometry	PLCζ expression showed a positive correlation with fertilization rate and a negative correlation with the percentage of DNA fragmentation, while no significant relationship was found between PLCζ and embryo quality or pregnancy rate.
[29]	Expression profile	Patients with varicocele (N = 35)	Fresh	Real-time PCR and Western blot	The mean relative expression of PLCζ was significantly lower in individuals with varicocele compared to fertile men at both transcription and translation levels. The percentage of DNA fragmentation was significantly higher in infertile men with varicocele compared to fertile men.
[30]	Expression profile	Normozoospermic men (N = 10)	Fresh	Flow cytometry	The proportion of sperm-expressing weak or zero levels of PLCζ were significantly reduced after DGC processing. There were no significant differences among processed DGC, capacitated and acrosome-reacted samples.
[31]	Expression profile, localization patterns	Normozoospermic (N = 13)	Fresh	Flow cytometry	The number of PLCζ-positive spermatozoa was significantly low in the Zeta method, but the intensity of PLCζ protein in such spermatozoa was significantly higher than DGC procedure.
[32]	Expression profile, protein level	Fertilization Failure (N = 15)	Fresh	Immunofluorescence	The mean percentage of PLCζ positive sperm and level of PLCζ protein were significantly lower in FF group compared to the control group.
[33]	Expression profile, protein level	Infertile Patients (N = 43)	Fresh	Flow cytometry	Quantitative analyses showed no significant difference among the low and high fertilization groups when PLCζ ratio or mean fluorescent intensity was considered. No correlation was found between pregnancy rates and PLCζ quantity. None of the total fertilization failure cases were lack of PLCζ.
[34]	Expression profile, localization patterns	Normozoospermic (N = 32)	Fresh/Frozen	Immunofluorescence	The presence of PLCζ on spermatozoa was decreased after freezing–thawing procedures. The percentage of spermatozoa exhibiting PLCζ at post-acrosomal position was significantly greater before freezing while no significant difference was seen for the percentage of spermatozoa exhibiting PLCζ at equatorial position.

**Table 2:** Studies on the gene mutation implicated in failed fertilization.

Ref.	PLCζ Features	Population	PLCZ Identification	Gene Mutation	Main Findings
[11]	Mutation identification	Recurrent ICSI failure or abnormal sperm morphology (N = 9)	Direct sequencing, mini sequencing	PLCζ ^H233L^ and PLCζ ^H398P^	PLCζ ^H233L^ is predicted to disrupt local protein interaction. Both PLCζ ^H233L^ and PLCζ ^H398P^ mutations exist on distinct parental chromosomes, the former inherited from the patient’s mother and the latter from his father.
[12]	Mutation identification, expression profile	Total or partial FF (N = 37)	Immunofluorescence, Western blot, Sanger sequencing	Five single-nucleotide missense mutations: p.I120M, p.R197H, p.L224P, p.H233L, and p.S500 L. A frameshift variant p.V326K fs * 25	Six different mutations were identified in FF patients, and all were found in heterozygosis. PLCZ1 mutations were found in high frequency in patients presenting OAF (54.55%) compared to the no-OAF group (6.67%). None of the controls presented with mutation in the PLCZ1 coding sequence.
[35]	Localization pattern in HEK293T cells	Recurrent ICSI failure (N = 1)	Confocal microscopy, microsequencing	PLCζ ^H233L^ and PLCζ ^H398P^	Both PLCζ ^H233L^ and PLCζ ^H398P^ mutations are present on different alleles and do not alter PLCζ localization in HEK293T cells.
[36]	Mutation identification	TTF (N = 2)	Whole exomic sequencing	Missense homozygote mutation in PLCZ1, c.1465A > T; p.Ile489Phe	The mutation is deleterious, leading to the absence of the protein in sperm, mislocalization of the protein when injected in GV and MII oocytes, highly abnormal Ca^2+^ transients, and early embryonic arrest.
[37]	Expression profile	Patients with total, low, and high fertilization rates and globozoospermic patients (N = 59)	Real time PCR, Western blot	CAPZA3 and PLCζ genes	The results revealed a significant correlation between the expression of CAPZA3 and PLCζ genes. Individuals with low expression of both genes presented low fertilization rates.
[38]	Mutation identification, AOA	Recurrent fertilization failure (N = 4)	Whole-exome sequencing, Sanger sequencing, Western blot	Homozygous nonsense mutation c.588C > A (p.Cys196*), homozygous missense mutation c.590G > A (p.Arg197His); a heterozygous mutation c.588C > A (p.Cys196*) and the missense heterozygous mutation c.1259C > T (p.Pro420Leu), compound heterozygous frameshift mutations c.972_973delAG (p.Thr324fs) and c.1234delA (p.Arg412fs)	Western blot showed that missense mutations decreased the level of PLCZ1, and that nonsense or frameshift mutations resulted in undetectable or truncated proteins. The oocyte activation ability was significantly reduced by these mutations. AOA helps to achieve a pregnancy.
[39]	Mutation identification	Recurrent partial/total fertilization failure (N = 2)	Sanger Sequencing	Compound heterozygous mutations c.1259C > T (p.P420L) and c.1733T > C (p.M578T) in the PLCZ1 gene; homozygous mutation c.1727T > C (p.L576P)	The compound heterozygous mutations c.1259C > T (p.P420L) and c.1733T > C (p.M578T) in the PLCZ1 gene were identified in a patient with TFF while the homozygous mutation c.1727T > C (p.L576P) was identified in a man with partial fertilization failure. These mutations were absent in the control cohort and in the databases. All mutant amino acids are located in key domains and are predicted to impair hydrolytic activity and lead to PLCZ1 dysfunction.
[40]	Mutation identification	Recurrent ICSI failure (N = 1)	Single-cell trio-seq sequencing, whole genome sequencing, Sanger sequencing, Western blot	Missense homozygous mutation in PLCζ, c.1658 G > C; p. R553P	PLCζ, c.1658 G > C mutation does not affect the production of the corresponding protein in sperm. However, microinjection of the mRNA transcribed from the PLCζ R553P mutation gene failed to trigger oocyte activation and the subsequent embryo development.
[41]	Mutation identification, localization patterns, AOA	TFF and poor ICSI fertilization (N = 10)	Whole-exome sequencing, immunofluorescence	A nonsense variation, c.C588A (p.C196X), two missense variations, c.T1048C (p.S350P) and c.C736T (p.L246F).	The three novel homozygous variations in the PLCZ1 gene are predicted to modify its secondary structure, impairing its hydrolytic activity. These variations in PLCZ1 led to poor or failed fertilization that could be overcome with ICSI-AOA.
[42]	Mutation identification, AOA	FF or poor fertilization (N = 14)	Sanger sequencing, Western blot	Nonsense mutation c.588C > A (p. C196*) and missense mutation c.830T > C (p.L277P), 3 bp in-frame deletion c.1129_1131delAAT (p.N377del), missense mutation c.1733T > C (p.M578T), splicing mutation c.570 + 1G > T and missense mutation c.1344A > T (p.K448N)	In five families, six new PLCZ1 mutations and one reported mutation were found. Western blot analysis showed no PLCZ1 protein in affected patients’ semen. The injection of wild-type PLCZ1 cRNA induced pronuclear formation, while the microinjection of mutant PLCZ1 cRNA did not activate oocytes to induce nuclear formation. AOA treatment successfully rescued fertilization failure in four patients.
[43]	Mutation identification, AOA	FF (N = 4)	Sanger sequencing, Western blot, immunofluorescence	c.588C > A (p.Cys196Ter)	We identified a novel homozygous PLCZ1 nonsense mutation, c.588C > A (p.Cys196Ter) in an infertile man from a consanguineous family. This results in the non-production of the protein; The use of AOA resulted in an increased rate of normal fertilization.
[44]	Expression level	Severe oligozoospermic patients (N = 8)	TaqMan low density array	44 genes	The transcript levels of 21 genes important for spermatogenesis and early preimplantation development, were significantly decreased in the severe oligozoospermic group. Among them, mRNA of AKAP4 and PTK7 was greatly reduced and the transcripts of PLCζ and POU5F1 were not detected in patients with severe oligozoospermia.
[45]	Mutation identification, expression level	Asthenoteratozoospermic patients (N = 105)	Quantitative RT-PCR, Western blot, immunofluorescence	c.1237A > T: p.Ile413Phe in SLO3	A homozygous missense variant (c.1237A > T: p.Ile413Phe) in the sperm-specific SLO3 in one Chinese patient was identified. The levels of SLO3 mRNA and protein in spermatozoa were reduced. Sperm of this individual exhibited acrosome hypoplasia, disruption of the mitochondrial sheath, coiled tails, and motility defects. Furthermore, the acrosome reaction, mitochondrial membrane potential, and membrane potential during capacitation were also afflicted. The levels of PLCζ1 were significantly reduced.
[46]	Expression profile, AOA	Globozoospermic patient (N = 1)	Immunofluorescence, Western blot, liquid chromatography–mass spectrometry/mass spectrometry, coimmunoprecipitation analyses	Homozygous missense variant (NM_001130445.3: c.3671G > A; p.R1224Q) in the SSFA2	This study revealed that SSFA2 plays an important role in acrosome formation, and the homozygous c.3671G > A loss-of-function variant in SSFA2 caused globozoospermia. AOA rescued the oocyte activation failure for the patient with the SSFA2 variant.

**Table 3:** Studies on PLCζ in patients with globozoospermia.

Ref.	PLCζ Features	Population	Sample	PLCζ Identification	Main Findings
[2]	Expression profile, genetics, AOA	Globozoospermic patients (N = 14)	Fresh	Immunofluorescence	PLCζ was detected in men with the partial form of globozoospermia, whereas it was not detected in those with the complete form. The PLCZ1 gene was mutated and underexpressed in the complete form of globozoospermia.
[47]	Expression profile, protein levels	Globozoospermic patients (N = 12)	Fresh	Quantitative real-time PCR analysis and Western blot	Levels of PLCζ mRNA in the spermatozoa of fertile men were significantly higher than globozoospermic men. At the protein level, expressions of these factors were low in globozoospermic individuals. High fertilization and pregnancy rates were achieved following ICSI-AOA.
[48]	Expression profile, protein levels	Globozoospermic patients (N = 21)	Fresh	Real time polymerase chain reaction (qPCR) and Western blot	Expression of PLCζ was significantly reduced at RNA and protein levels in globozoospermic patients compared to fertile men.
[49]	Expression profile, protein levels	Globozoospermic patients with DPY19L2 deletion (N = 32)	Fresh	Quantitative real-time PCR analysis and Western blot	The relative expression of PLCζ at RNA and protein levels in globozoospermic men with DPY19L2 deletion was significantly lower compared with fertile men. The fertilization rate in globozoospermic couples following ICSI-AOA was significantly lower. Implantation and pregnancy rates were not jeopardized by DPY19L2 deletion in these couples.
[50]	Expression profile, localization patterns, level	Globozoospermic patients (N = 3)	Fresh	Quantitative immunofluorescence	Completely round-headed globozoospermic sperm were either devoid of PLCz immunofluorescence or exhibited an abnormal, punctate pattern of PLCz localization. Most sperm with an acrosomal bud exhibited punctate patterns of PLCz localization within the sperm head. MSOME selects spermatozoa with a higher total level and proportion of spermatozoa exhibiting PLCζ.

**Table 4:** Studies on the AOA procedure in patients with PLCζ deficiency.

Ref.	PLCζ Features	Population	Sample	PLCζ Identification	Main Findings
[3]	AOA and clinical outcomes	Poor fertilization/FF (N = 76)	Fresh	Immunofluorescence, MOAT	For couples with oocyte-related OAD, successful fertilization, term pregnancies, and deliveries were achieved with an adjusted superovulation protocol. In couples with a sperm-related OAD, as determined via PLCζ assay, assisted gamete treatment was successful.
[51]	AOA and clinical outcomes	Infertile patients with PLCζ deficient (N = 220)	Fresh	Immunofluorescence	The fertilization rate was significantly lower in the group without AOA compared to the control group. Cleavage and embryo quality scores were not substantially different between groups of control, with and without AOA treatment.
[52]	Expression profile, localization patterns, AOA, and clinical outcomes	Teratozoospermic men, TFF, low fertilization rate (N = 43)	Fresh	Immunofluorescence	Compared with fertile controls, infertile males had significantly lower levels of PLCζ and/or a significantly lower proportion of sperm exhibiting PLCζ. In men with a significant PLCζ reduction in both parameters, the fertilization and clinical pregnancies rate improved significantly after AOA treatment.
[53]	Measurement of fertilizing and calcium-releasing ability	Recurrent partial hydatidiform mole pregnancy (N = 1)	Fresh	Immunofluorescence, MOAT	Sperm that previously provoked recurrent partial hydatidiform mole pregnancies due to dispermic fertilization are not able to trigger the normal pattern of Ca^2+^ oscillations in oocytes. In subsequent ICSI cycles, fertilization failure was overcome with AOA treatment which led to normal deliveries.

**Table 5:** Studies on new clinical approaches in men with PLCζ impairment.

Ref.	PLCζ Features	PLCζ Identification	Treatment	Main Findings/Function
[7]	Review on the application of PLCζ in diagnostic and therapeutic medicine	NGS	hrPLCζ	NGS represents a more sensitive and less time-consuming method for screening PLCζ genetic variants and carefully designed promoters. The most outstanding priority is to synthesize a pure hrPLCZ1 with which to generate a monoclonal antibody and protein crystals.
[13]	Review on the effectiveness of oocyte activation methods	NA	AOA, hrPLCζ	The use of calcium ionophore directly increases [Ca^2+^] but it can have a potential cytotoxic or mutagenic adverse effect on eggs and embryos. The PLCZ1 RNA injection can induce an optimal pattern of Ca^2+^ oscillations at fertilization, leading to the parthenogenetic development of blastocysts. Nevertheless, the adverse effect of RNA injection on human oocytes is still unclear.
[14]	Review of the safety and efficiency of AOA	NA	AOA	Contradictory studies on the safety and efficacy of AOA do not yet allow for the establishment of AOA as standard practice in the clinic. The main scientific concern is the non-physiological method of Ca^2+^ release mediated by most AOA agents, coupled with a lack of holistic understanding regarding the physiological mechanism(s) underlying Ca^2+^ release at oocyte activation.
[16]	Review on the function of PLCζ and new approach focusing on its clinical applicability	NA	hrPLCζ	ICSI failure may be related to impaired PLCζ activity. Microinjection of recombinant human PLCζ to human oocytes after ICSI fertilization failure may trigger Ca^2+^ oscillations and achieve successful fertilization.
[54]	Review of genetic causes of FF, the use of PLCZ1 as a diagnostic marker or therapeutic molecule treatments	MOAT, MOCA, HOCA	hrPLCζ	Heterologous (MOAT and MOCA) and homologous ICSI tests (HOCA) have a high predictive potential for identifying sperm-related deficiencies, but they are technically difficult and require animal facilities and specialized equipment. The injection of human rPLCZ1 protein results in multiple Ca^+2^ oscillations resembling the physiological calcium. However, it is not yet commercially available. In the meantime, AOA with calcium ionophores remains the best strategy.
[55]	Calcium oscillation mechanism	Particle image velocimetry (PIV) and Ca^2+^ sensitive fluorescent dye	hrPLCζ	Microinjection of PLCζ cRNA into human oocytes that had failed to fertilize after ICSI resulted in the appearance of prolonged Ca^2+^ oscillations. Ca^2+^ concentration change was accompanied by a small, coordinated movement of the cytoplasm that could be detected using particle image velocimetry (PIV) analysis.
[56]	Pattern of Ca^2+^ oscillation with different activation methods	PCR technique	hrPLCζ	The pattern of Ca^2+^ oscillations after PLCZ1 RNA injection exhibited similar characteristics to that after ICSI treatment. Human PLCZ1 RNA is a better therapeutic agent to rescue human oocytes from failed activation, leading to normal and efficient development.
[57]	Expression levels of PLCZ1 with microfluidics sperm selection	Reverse transcription-polymerase chain reaction (RT-PCR)	Sperm selection through DGC and microfluidics	The RT-PCR results showed that there was a significant increase in the expression of PLCZ1 and TNP1 genes in the sperm of groups that were selected using microfluidics sperm selection compared to the group that underwent the density gradient centrifugation method.

## Data Availability

Data are contained within the article.
